# Postural Evaluation in Young Healthy Adults through a Digital and Reproducible Method

**DOI:** 10.3390/jfmk7040098

**Published:** 2022-10-28

**Authors:** Bruno Trovato, Federico Roggio, Martina Sortino, Marta Zanghì, Luca Petrigna, Rosario Giuffrida, Giuseppe Musumeci

**Affiliations:** 1 Department of Biomedical and Biotechnological Sciences, Section of Anatomy, Histology and Movement Science, School of Medicine, University of Catania, Via S. Sofia No. 97, 95123 Catania, Italy; 2 Sport and Exercise Sciences Research Unit, Department of Psychology, Educational Science and Human Movement, University of Palermo, Via Giovanni Pascoli 6, 90144 Palermo, Italy; 3 Department of Biomedical and Biotechnological Sciences, Section of Physiology, School of Medicine, University of Catania, 95125 Catania, Italy; 4 Research Center on Motor Activities (CRAM), University of Catania, Via S. Sofia No. 97, 95123 Catania, Italy; 5 Department of Biology, Sbarro Institute for Cancer Research and Molecular Medicine, College of Science and Technology, Temple University, Philadelphia, PA 19122, USA

**Keywords:** posture, reproducibility, mobile app, movement, kinesiology

## Abstract

Different tools for the assessment of posture exist, from the simplest and cheap plumb line to complex, expensive, 3D-marker-based systems. The aim of this study is to present digital postural normative data of young adults collected through a mobile app to expand the possibilities of digital postural evaluation. A sample of 100 healthy volunteers, 50 males and 50 females, was analyzed with the mobile app Apecs-AI Posture Evaluation and Correction System^®^ (Apecs). The Student’s *t*-test evaluated differences between gender to highlight if the digital posture evaluation may differ between groups. A significant difference was present in the anterior coronal plane for axillary alignment (*p* = 0.04), trunk inclination (*p* = 0.03), and knee alignment (*p* = 0.01). Head inclination (*p* = 0.04), tibia shift (*p* = 0.01), and foot angle (*p* < 0.001) presented significant differences in the sagittal plane, while there were no significant differences in the posterior coronal plane. The intraclass correlation coefficient (ICC) was considered to evaluate reproducibility. Thirteen parameters out of twenty-two provided an ICC > 0.90, three provided an ICC > 0.60, and six variables did not meet the cut-off criteria. The results highlight that digital posture analysis of healthy individuals may present slight differences related to gender. Additionally, the mobile app showed good reproducibility according to ICC. Digital postural assessment with Apecs could represent a quick method for preventing screening in the general population. Therefore, clinicians should consider this app’s worth as an auxiliary posture evaluation tool.

## 1. Introduction

Posture is defined as the position acquired by the human body in various situations, opposed to the force of gravity and adapting to different environments [1]. Moreover, posture is essential for maintaining postural balance both in static and dynamic conditions. Remaining in a non-ergonomic position for an extended period can predispose people to manifest musculoskeletal pain [2]; thus, assuming good postures is considered necessary for general health at both a musculoskeletal and psychological level [3]. Nowadays, the evaluation of human posture is performed consistently in healthcare clinics and fitness centers, considering that postural misalignments can cause individuals to manifest headaches, lower back pain, neck pain, neurological pathologies, and a reduction in overall psychological well-being [4]. Currently, the literature does not provide any gold standard procedure for postural assessment. The types of exams employed can vary from a visual evaluation with goniometers and plumb lines to motion capture systems, such as Vicon, for dynamic evaluation and 3D camera infrared systems, such as rasterstereography, for static evaluation [5]. Regarding the feasibility of using a markerless system to assess human posture, rasterstereography is a system that generates a 3D model of the spine by calculating specific deformities and analyzing the convexity and concavity of the spine [6]. It is commonly used to investigate the presence of scoliosis and is considered reliable for the assessment of parameters such as pelvic obliquity, thoracic kyphosis, and lumbar lordosis angles [7,8]. However, this system has a high cost, and it is difficult to implement in postural screening for the general population. Other valid tools such as inertial measurement units (e.g., accelerometers, magneto inertial units) are also employed in the field of postural evaluation for the assessment of the thoracic kyphosis and the lumbar lordosis angles [9] and also for gait and balance assessment [10]. 

All the available methods for evaluating posture present some biases or disadvantages. The visual evaluation with a plumb line is cheap, but it requires specialized personnel, is prone to bias, and lacks scientific validation [11]. The use of goniometers is feasible for the measurement of the range of motion and angles of different joints with good reliability [12]; it has a low cost and is easy to perform, although it presents some methodological issues when assessing postural deviations [13], and it is only considered useful for one postural variable examination at the time [5]. Marker-based advanced technologies that can provide highly accurate data on joint angles and translations are potentially available for clinicians; however, these evaluation systems are too expensive for the average clinic, and often they are employed for research purposes only [14]. 

In this heterogeneous scenario regarding the available postural evaluation tools, the advancement in image-based technologies will come in handy for clinicians and researchers who want to find a postural assessment system with good reproducibility and an affordable cost. Tablet and phone apps for postural evaluation can fill this gap, with different postural apps demonstrating promising results in the evaluation of the frontal plane [15], standing posture [13], angulation variables [14], and head shift in sagittal and frontal planes [4]; however, the literature is insufficient to confirm the quality of these methods. Considering that the complete visual evaluation of body posture with goniometers and a plumb line can be long and not free from biases, and taking into account the high costs of 3D systems, the use of a mobile app could represent a quick, safe, and accurate method for researchers and clinicians to quantitatively evaluate general posture. Moreover, laboratory tests are often more expensive than field-based ones [16], and adopting a mobile, affordable tool for postural assessment could benefit the primary prevention of musculoskeletal disorders of the spine. The aim of this study is to present normative data about digital posture evaluation collected through a mobile app Apecs and, moreover, to evaluate the reproducibility. 

## 2. Materials and Methods

### 2.1. Participants

We recruited and evaluated a sample of 100 healthy volunteers, 50 males and 50 females, with a mean age of 23.4 (standard deviation (SD) ± 6.2) years. Prior to testing, all participants were informed about the study procedure, risks, and benefits and provided written, informed consent to participate in the study and for us to use their data. The study followed the Helsinki Declaration principles and was approved by the University of Catania (protocol no.: CRAM-017-2020, 16 March 2020).

Exclusion criteria comprised: past or current major musculoskeletal injuries, spine pathology, and neurological pathologies. All the participants selected after the oral interview underwent a static postural evaluation provided by expert clinician (experience of 7 years) to confirm their eligibility for the study. 

### 2.2. Study Design

**Figure 1:**
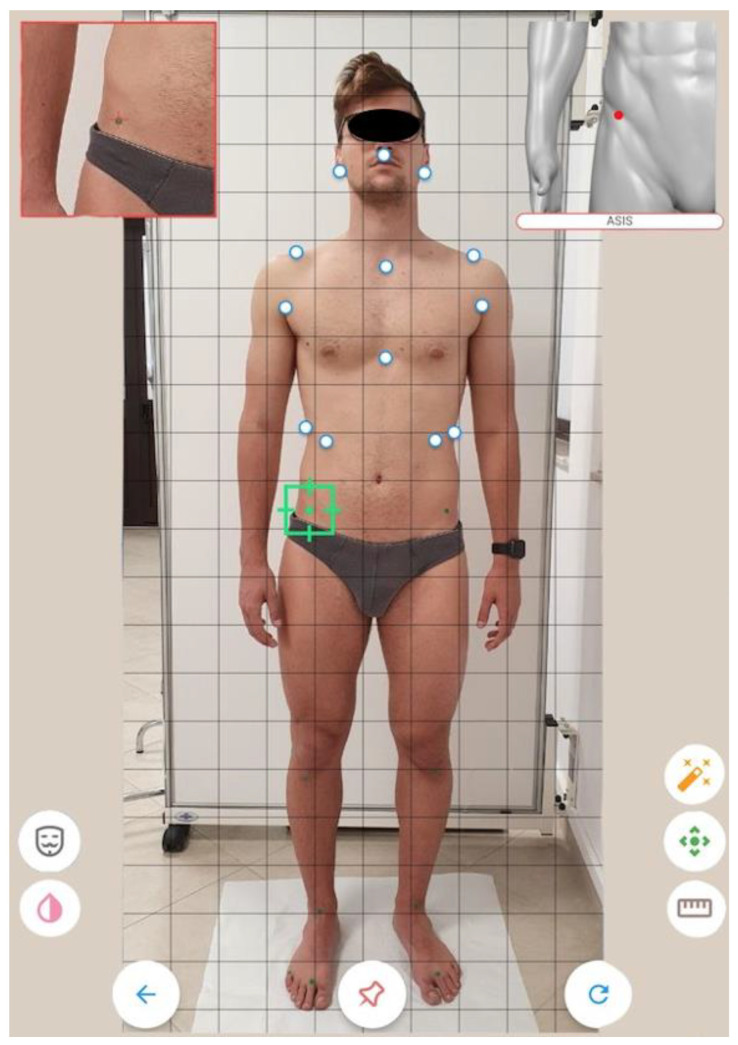
Landmarks positioning.

The evaluation took 30 min per participant at the University Laboratories and consisted of evaluating their health status and history of previous conditions that could meet the exclusion criteria. After the screening process, participants were always asked to attend the laboratory at the same time (between 10:00 a.m. and 12:00 p.m.). The postural evaluation was performed by three different operators with similar experience in postural analysis (3 to 4 years of experience). The mobile app Apecs-AI Posture Evaluation and Correction System^®^ (New Body Technologies SAS, Grenoble, France) (Apecs app) was used to acquire the images of the participants in standing position. The participants were asked to dress in minimal clothing, shorts for men and shorts and bras for women, to minimize biases relating to wrong landmark positioning during the postural analysis; for the same reason, markers were placed by expert clinicians on the body of the participants in correspondence to the app’s predetermined landmarks. Four pictures were captured, one for the anterior coronal plane, one for the posterior coronal plane, and two for the sagittal plane (left and right). Participants were instructed to place their feet at the same width as the shoulders (Figure 1). 

**Figure 2:**
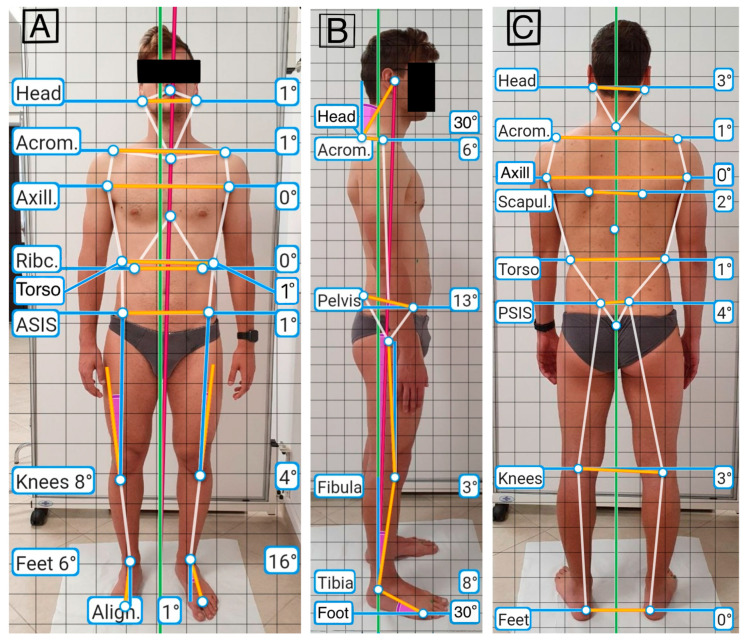
Evaluation of the anterior coronal plane (**A**); of the sagittal plane (**B**); of the posterior coronal plane (**C**).

To avoid any wrong camera leveling during the image acquisition, the app’s interface shows a target that becomes green when the camera is leveled. After the picture is acquired, the app immediately steers the user to crop the image at the individual’s head and feet to minimize inconsistency in the proportion of different images. The Apecs app uses standardized digital landmarks and anatomical angles from one to four pictures, depending on the number of variables of interest to the investigation. The app calculates 24 postural variables from the predetermined anatomical markers in the three planes of the space examined. Figure 2 shows the points evaluated in the anterior coronal plane (a), the sagittal plane (b), and the posterior coronal plane (c).

After the cropping phase, the app drives the user to position the digital markers, fostering this process with examples of the proper positioning with images. Table 1 shows all the anatomical landmarks taken into consideration by the app for calculating the postural variables.

### 2.3. Statistical Analysis

Data analysis comprised descriptive statistics to present the mean and standard deviation of the whole sample divided by gender. Inferential statistics comprised the Shapiro–Wilk test to assess the data distribution; the Student’s *t*-test was used to compare means between the male and female groups; statistical significance was set at *p* ≤ 0.05. Cohen’s effect size (d) was applied to identify meaningful differences between the groups. Based on Cohen’s criteria, d = 0.80 (absolute value) was considered a large effect size, and d = 0.50 (absolute value) was considered a medium effect size. Post hoc power calculations were performed with G*Power v.3.1. Three qualified examiners were selected to perform the positioning of the markers and the postural analysis in the two different parts of the day to assess the reproducibility of the app. The two-way mixed effect for absolute agreement was the model for calculating the intraclass correlation coefficient (ICC) for inter-rater agreement. The cut-off values for reproducibility based on a 95% confidence interval of the ICC estimate were <0.5 (poor), between 0.5 and 0.75 (moderate), between 0.75 and 0.9 (good), and >0.9 (excellent) [17]. All the statistical analyses were performed with R Project for Statistical Computing (Vienna, Austria). 

## 3. Results

Anthropometric measurements were taken for each subject and grouped by gender, with a mean male height of 175 (SD ± 5.6) cm, a mean female height of 164.6 (SD ± 6.5) cm, and a mean male weight of 75.5 (SD ± 8.8) kg and a mean female weight of 58.13 (SD ± 7.41) kg. 

The post hoc power calculation analysis with G*Power 3.1 returned a statistical power of 0.696 for our sample. The analysis of the digital anatomical landmarks collected with the Apecs app and the ICC values are presented in Table 2. The Student’s *t*-test statistically indicated differences in the postural evaluation performed by the mobile app Apecs between males and females for specific variables. The postural variables with significant differences between male and female groups in the anterior coronal plane were axillary alignment (*p* = 0.04), trunk inclination (*p* = 0.03), and knee alignment (*p* = 0.01). The female group presented more body inclination to the right than men, more trunk inclination, and a wider knee angle in the anterior coronal plane. The male group showed the worst results for the axillary alignment, which resulted in greater deviation from the ideal alignment than that of the female group. In the sagittal plane, statistically significant differences were found for head inclination (*p* = 0.04), tibia shift (*p* = 0.01), and foot angle (*p* < 0.001). The head of the female group was more significantly shifted from the ideal alignment compared to that of the male group and also showed a more accentuated anterior tibial shift. Instead, the male group presented a wider foot angle than the female group. No statistically significant differences were found between groups for the evaluation of the posterior coronal plane. According to Cohen’s d, there was a small effect size only for ribcage tilt (d = −0.35) in the anterior coronal plane, for head alignment in the sagittal plane (d = −0.38), and a large effect size for knee angle in the anterior coronal plane (d = −0.89), tibia shift in the sagittal plane (d = −0.95), and foot angle in the sagittal plane (d = 1.6).

**Figure 3:**
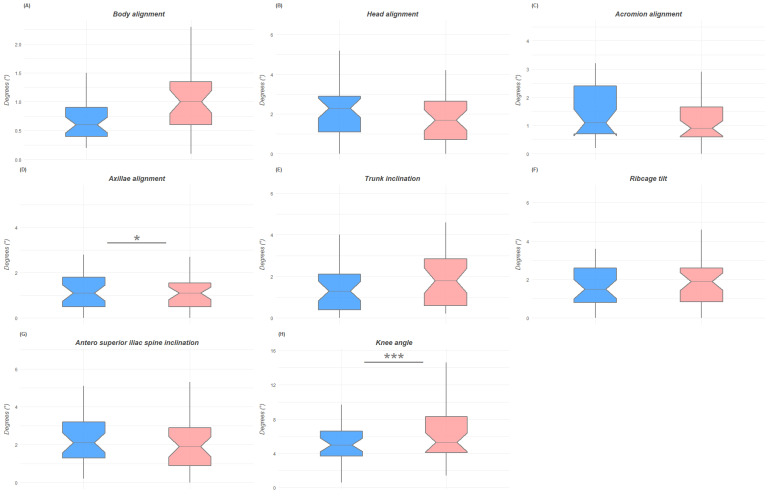
Box plots of the differences between male and female groups in the anterior coronal plane with indication of significance. Figure (**A**) is for body alignment; figure (**B**) is for head alignment, figure (**C**) is of acromion alignment, figure (**D**) is for axillae alignment, figure (**E**) is for trunk inclination, figure (**F**) is for ribcage tilt, figure (**G**) is for antero superior iliac spine inclination, figure (**H**) is for knee angle. *: *p* < 0.05; ***: *p* < 0.001.

**Figure 4:**
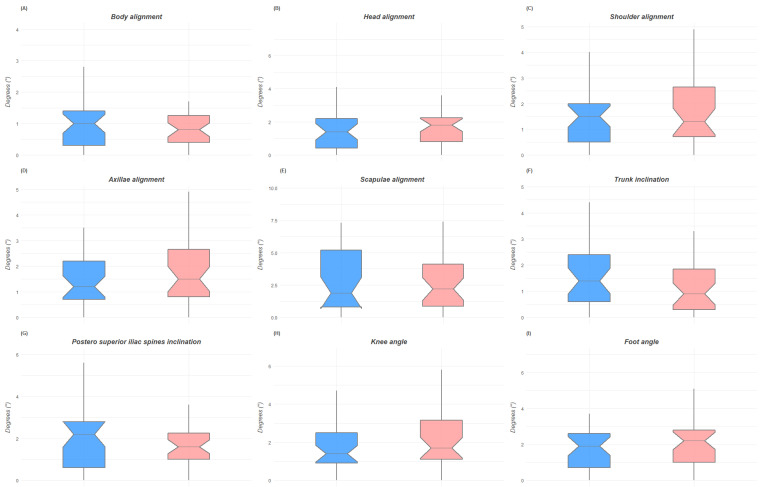
Box plots of the differences between male and female groups in the posterior coronal plane. Figure (**A**) is for body alignment; figure (**B**) is for head alignment, figure (**C**) is of shoulder alignment, figure (**D**) is for axillae alignment, figure (**E**) is for scapulae alignment, figure (**F**) is for trunk inclination, figure (**G**) is for postero superior iliac spine inclination, figure (**H**) is for knee angle, figure (**I**) is for foot angle.

**Figure 5:**
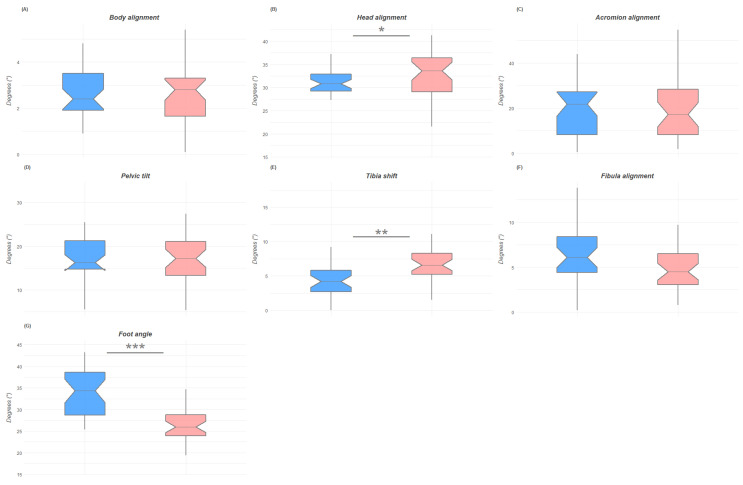
Box plots of the postural differences between male and female groups in the sagittal plane with indication of significance. Figure (**A**) is for body alignment; figure (**B**) is for head alignment, figure (**C**) is of acromion alignment, figure (**D**) is pelvic tilt, figure (**E**) is for tibia shift, figure (**F**) is for fibula alignment, figure (**G**) is for foot angle. *: *p* < 0.05; **: *p* < 0.01; ***: *p* < 0.001.

Figure 3, Figure 4 and Figure 5 show the box plots for gender differences in the three space planes.

The ICC showed promising results for inter-rater reproducibility, with values > 0.90 for thirteen out of the twenty-two postural variables examined and >0.60 for the other three variables; only six variables did not meet the cut-off criteria required to be considered reliable. Table 2 shows the ICC for the postural variables evaluated.

## 4. Discussion

This study aimed to present normative data about the digital posture evaluation of healthy young adults performed by the mobile app Apecs and to evaluate its reproducibility. The first finding was that the app is sensible to postural variation, considering that it was capable of detecting postural differences between males and females. The second finding of the study was that this mobile app presents a good inter-rater reproducibility for all the postural variables examined except for head alignment, trunk inclination and axillae alignment in the anterior and posterior coronal plane, and acromion alignment in the sagittal plane.

The Apecs app has already been used for research purposes to evaluate postural behaviors related to specific ergonomic studies’ work [12] and to evaluate body segment angles in subjects with adolescent idiopathic scoliosis [18]. However, the studies mentioned above had small samples; the first used the app only to compare their sample’s posture at rest and during working activity, and the second only evaluated angles in the frontal and sagittal plane. Hence, to the best of our knowledge, this is the first study that employs the mobile app Apecs to evaluate global posture, providing normative data and assessing its reproducibility as a posture evaluation tool. 

The sample in this study was composed of 100 participants equally distributed between males and females, and the Apecs mobile app was capable of detecting postural differences when present. It emerged from the postural analysis of the anterior coronal plane that females presented a wider knee angle; this could be due to the overall increased knee laxity and reduced stiffness in females compared to males [19]. In a previous study by Raine et al. [20], no sex differences were found for head inclination on the sagittal plane; conversely, we found that the head inclination was more accentuated in the female group compared to the male group. However, Raine et al.’s study dates from 1997, and they considered an older sample size. These observations may be the cause of the differences compared to our study. Iacob et al. [21] analyzed the posture of a sample of people with malocclusion through the PostureScreen^®^ mobile app, comparing it with a healthy sample. We found a difference between our postural data gathered with Apecs and those reported by Iacob et al. for the same variable analyzed. These authors found, on the frontal plane, a head alignment in their sample of 3.86° ± 2.45, a shoulder alignment of 1 ± 0.97, and a hip deviation of 1.42 ± 1.28, while, for the same variable, we reported a head alignment of 2.7 ± 1.5, a shoulder alignment of 1.5 ± 1.2, and postero superior spine inclination of 1.9° ± 1.4. The differences in the postural evaluation between the two apps might be due to the differences in the samples considering that the control group of young healthy young adults investigated by Iacob et al. was composed of only 14 people, and they were almost exclusively females.

We found a statistically significant difference in the sagittal plane for foot angle, with the male group presenting higher values; this finding could be related to the generally bigger size of the foot anthropometrics of males [22]. In the anterior and posterior coronal planes, we did not find any statistically significant difference between gender for foot posture parameters, in line with previous studies [23,24]. 

The reproducibility analysis of Apecs showed excellent results for all the variables examined on the sagittal plane except for the acromion alignment. The marker placed on the acromion was not clearly visible during the positioning of the digital marker in this plane of space, making it difficult to evaluate with consistency among raters. The same issue occurred in the posterior coronal plane for the trunk inclination, where the app asks raters to identify “the most intended point of the trunk”, which was not easy to replicate for the raters. Interestingly, the two less reliable measures in the anterior coronal plane were the axillae alignment and the trunk inclination, indicating that this mobile app should be carefully considered when a precise measure of these variables is needed. Accordingly, with what was stated by Szucs et al. [14] when evaluating the PostureScreen^®^ mobile app, we suggest that the quality of the evaluation is higher when markers are placed on the subject and are clearly visible during the positioning of the digital markers; however, neither the Apecs manufacturer nor the PostureScreen mobile one specifies this in their instruction for postural analysis.

The current study presents some limitations. First, we considered a sample composed exclusively of young adults, so we could not assess whether the Apecs app is a feasible tool to employ in the postural evaluation of pediatric and elderly populations. Second, all the individuals in the sample were healthy, thus, these results should be carefully interpreted when examining individuals with pathologies that influence the musculoskeletal system. Third, we did not compare measures collected with Apecs with those collected by postural gold standard instruments to assess the validity of the app. Further studies should investigate the validity of Apecs as a reliable postural assessment tool, comparing it with rasterstereography or marker-based systems. However, these normative data may help those involved in the analysis of postural alterations as a comparative standard with a healthy sample. Finally, the digital landmark positioning accomplished with the app may be challenging for less experienced users and might change the evaluation results.

## 5. Conclusions

The mobile postural app Apecs demonstrated good reproducibility for most of the postural variables analyzed and could detect postural differences between males and females when present. The app was easy to use for all the raters, from the more experienced to the less experienced ones, indicating that Apecs could be a cheap and feasible good alternative to more expensive postural assessment devices for researchers and clinicians. However, trunk inclination and axillae alignment were unreliable in all the planes of space where they were evaluated, and head alignment was reliable only in the sagittal plane. Clinicians should be aware of this issue when using Apecs and carefully predetermine the landmark positioning and digital identification during the analysis to minimize the possibilities of errors for the postural variables not clearly described by the Apecs manufacturer. In conclusion, the Apecs app could be a potentially useful tool for clinicians and researchers to implement in the preventive care of postural disorders given its ease of use and cheap costs.

## Figures and Tables

**Table 1:** Anatomic landmarks and postural variables studied with Apecs.

Plane of the Space	Anatomical Landmarks	Postural Variables
Anterior coronal	Acromion Anterior axillary folds Anterior superior iliac spine Jugular notch Lobulus auriculae Lowest point of costal margin Midpoint between malleoli Most intended point of the trunk Philtrum Second metatarsophalangeal joint Tibial tuberosity Xiphoid process	Body alignment Head alignment Acromion alignment Axillae alignment Trunk inclination Ribcage tilt Antero superior iliac spine inclination Knee angle
Posterior coronal	Lobulus auriculae C-7 vertebrae Acromion Anterior axillary folds Inferior angle of the scapula T-6 vertebrae Most intended point of the trunk Posterior superior iliac spine Superior end of intergluteal cleft Popliteal fossa Calcaneal tuberosity	Body alignment Head alignment Shoulder alignment Axillae alignment Scapulae alignment Trunk inclination Postero superior iliac spines Knee angle Foot angle
Sagittal	Tragus C-7 vertebrae Acromion Posterior superior iliac spine Greater trochanter Lateral joint line Lateral malleolus Head of the fifth metatarsal bone	Body alignment Head alignment Acromion alignment Pelvic tilt Tibia shift Fibula alignment Foot angle

**Table 2:** Description of group means and ICC of the postural variables analyzed.

	Postural Variables	Total	Males	Females	*t*-Test	ICC	Cohen d
		Mean ± SD	Mean ± SD	Mean ± SD			
Anterior coronal	Body alignment	0.9° ± 0.5	0.7° ± 0.4	1° ± 0.5	0.430	0.95	−0.54
	Head alignment	2° ± 1.4	2.2° ± 1.8	1.8° ± 1.4	0.989	0.51	0.25
	Acromion alignment	1.3° ± 1	1.4° ± 0.9	1.2° ± 1.1	0.423	0.91	0.17
	Axillae alignment	1.3° ± 1	1.4° ± 1.4	1.2° ± 0.8	0.044 *	0.25	0.16
	Trunk inclination	1.6° ± 1.2	1.4° ± 1.3	1.8° ± 1.1	0.462	0.44	−0.29
	Ribcage tilt	1.9° ± 1.6	1.7° ± 1.2	2.2° ± 1.8	0.039 *	0.93	−0.35
	ASIS inclination	2.3° ± 1.6	2.5° ± 1.7	1.5° ± 0.3	0.321	0.94	0.24
	Knee angle	6.2° ± 3.3	4.8° ± 2.9	7.5° ± 3.1	0.001 ***	0.93	−0.89
Posterior coronal	Body alignment	1° ± 0.8	1° ± 0.8	0.9° ± 0.8	0.717	0.84	0.07
	Head alignment	2.7° ± 1.5	1.6° ± 1.4	1.8° ± 1.5	0.652	0.30	−0.14
	Shoulder alignment	1.5° ± 1.2	1.4° ± 1.1	1.7° ± 1.4	0.444	0.93	−0.19
	Axillae alignment	1.6° ± 1.2	1.4° ± 1	1.7° ± 1.2	0.348	0.43	−0.26
	Scapulae alignment	2.7° ± 2.2	2.8° ± 2.3	2.7° ± 2.2	0.879	0.92	0.05
	Trunk inclination	1.4° ± 1.2	1.6° ± 1.2	1.3° ± 1.2	0.925	0.26	0.23
	PSIS inclination	1.9° ± 1.4	2° ± 1.6	1.8° ± 1.3	0.247	0.66	0.15
	Knee angle	1.9° ± 1.4	1.8° ± 1.2	2.1° ± 1.6	0.172	0.94	−0.23
	Foot angle	2.1° ± 1.6	1.9° ± 1.4	2.3° ± 1.8	0.151	0.75	−0.24
Sagittal	Body alignment	2.6° ± 1.2	2.6° ± 1.1	2.5° ± 1.3	0.691	0.94	0.1
	Head alignment	31.4° ± 5.4	30.3° ± 4.3	32.4° ± 6.2	0.047 *	0.91	−0.38
	Acromion alignment	19.6° ± 12.3	19.9° ± 12.1	19.4° ± 12.8	0.866	0.24	0.04
	Pelvic tilt	16.9° ± 5.7	16.6° ± 5.3	17.1° ± 6.1	0.763	0.94	−0.07
	Tibia shift	5.7° ± 3.3	4.2° ± 2.2	7.1° ± 3.5	0.017 **	0.91	−0.95
	Fibula alignment	5.5° ± 3	6.3° ± 3.1	4.8° ± 2.7	0.491	0.94	0.49
	Foot angle	29.8° ± 6	33.7° ± 26.2	26.2° ± 4.2	0.001 ***	0.93	1.6

ASIS: anterior superior iliac spines; PSIS: postero superior iliac spines. * *p*-value < 0.05; ** *p*-value < 0.01; *** *p*-value < 0.001.

## Data Availability

Data are available upon appropriate request.

## References

[1] Carini F., Mazzola M., Fici C., Palmeri S., Messina M., Damiani P., Tomasello G. (2017). Posture and posturology, anatomical and physiological profiles: Overview and current state of art. Acta Biomed..

[2] Rodrigues M.S., Leite R.D.V., Lelis C.M., Chaves T.C. (2017). Differences in ergonomic and workstation factors between computer office workers with and without reported musculoskeletal pain. Work.

[3] Harvey R.H., Peper E., Mason L., Joy M. (2020). Effect of Posture Feedback Training on Health. Appl. Psychophysiol. Biofeedback.

[4] Hopkins B.B., Vehrs P.R., Fellingham G.W., George J.D., Hager R., Ridge S.T. (2019). Validity and Reliability of Standing Posture Measurements Using a Mobile Application. J. Manip. Physiol. Ther..

[5] Fortin C., Feldman D.E., Cheriet F., Labelle H. (2011). Clinical methods for quantifying body segment posture: A literature review. Disabil. Rehabil..

[6] Roggio F., Ravalli S., Maugeri G., Bianco A., Palma A., Di Rosa M., Musumeci G. (2021). Technological advancements in the analysis of human motion and posture management through digital devices. World J. Orthop..

[7] Betsch M., Wild M., Jungbluth P., Hakimi M., Windolf J., Haex B., Horstmann T., Rapp W. (2011). Reliability and validity of 4D rasterstereography under dynamic conditions. Comput. Biol. Med..

[8] Molinaro L., Russo L., Cubelli F., Taborri J., Rossi S. Reliability analysis of an innovative technology for the assessment of spinal abnormalities. Proceedings of the 2022 IEEE International Symposium on Medical Measurements and Applications (MeMeA).

[9] Paloschi D., Bravi M., Schena E., Miccinilli S., Morrone M., Sterzi S., Saccomandi P., Massaroni C. (2021). Validation and Assessment of a Posture Measurement System with Magneto-Inertial Measurement Units. Sensors.

[10] Leirós-Rodríguez R., Romo-Pérez V., García-Soidán J.L., Soto-Rodríguez A. (2020). Identification of Body Balance Deterioration of Gait in Women Using Accelerometers. Sustainability.

[11] Tyson S.F., DeSouza L.H. (2003). A clinical model for the assessment of posture and balance in people with stroke. Disabil. Rehabil..

[12] Youdas J.W., Carey J.R., Garrett T.R. (1991). Reliability of measurements of cervical spine range of motion--comparison of three methods. Phys. Ther..

[13] Timurtaş E., Avcı E.E., Mate K., Karabacak N., Polat M.G., Demirbüken İ. (2021). A mobile application tool for standing posture analysis: Development, validity, and reliability. Ir. J. Med. Sci..

[14] Szucs K.A., Brown E.V.D. (2018). Rater reliability and construct validity of a mobile application for posture analysis. J. Phys. Ther. Sci..

[15] Moreira R., Fialho R., Teles A.S., Bordalo V., Vasconcelos S.S., Gouveia G.P.M., Bastos V.H., Teixeira S. (2022). A computer vision-based mobile tool for assessing human posture: A validation study. Comput. Methods Programs Biomed..

[16] Heyward V.H. (1992). Advanced fitness assessment and exercise prescription. Med. Sci. Sport. Exerc..

[17] Koo T.K., Li M.Y. (2016). A Guideline of Selecting and Reporting Intraclass Correlation Coefficients for Reliability Research. J. Chiropr. Med..

[18] Belli G., Toselli S., Latessa P.M., Mauro M. (2022). Evaluation of Self-Perceived Body Image in Adolescents with Mild Idiopathic Scoliosis. Eur. J. Investig. Health Psychol. Educ..

[19] Boguszewski D.V., Cheung E.C., Joshi N.B., Markolf K.L., McAllister D.R. (2015). Male-Female Differences in Knee Laxity and Stiffness: A Cadaveric Study. Am. J. Sports Med..

[20] Raine S., Twomey L.T. (1997). Head and shoulder posture variations in 160 asymptomatic women and men. Arch. Phys. Med. Rehabil..

[21] Iacob S.M.P., Chisnoiu A.M.P., Lascu L.M.P., Berar A.M.P., Studnicska D.M., Fluerasu M.I.P. (2020). Is PostureScreen® Mobile app an accurate tool for dentists to evaluate the correlation between malocclusion and posture?. Cranio.

[22] Zhao X., Tsujimoto T., Kim B., Katayama Y., Tanaka K. (2017). Characteristics of foot morphology and their relationship to gender, age, body mass index and bilateral asymmetry in Japanese adults. J. Back Musculoskelet Rehabil..

[23] Redmond A.C., Crane Y.Z., Menz H.B. (2008). Normative values for the Foot Posture Index. J. Foot Ankle Res..

[24] Gimunová M., Válková H., Kalina T., Vodička T. (2019). The relationship between body composition and foot posture index in Special Olympics athletes. Acta Bioeng. Biomech..

